# The Antarium: A Reconstructed Visual Reality Device for Ant Navigation Research

**DOI:** 10.3389/fnbeh.2020.599374

**Published:** 2020-11-10

**Authors:** Zoltán Kócsi, Trevor Murray, Hansjürgen Dahmen, Ajay Narendra, Jochen Zeil

**Affiliations:** ^1^Research School of Biology, Australian National University, Canberra, ACT, Australia; ^2^Department of Cognitive Neuroscience, University of Tübingen, Tübingen, Germany; ^3^Department of Biological Sciences, Macquarie University, Sydney, NSW, Australia

**Keywords:** visual navigation, virtual reality, reconstructed visual reality, ants, LED arena

## Abstract

We constructed a large projection device (the Antarium) with 20,000 UV-Blue-Green LEDs that allows us to present tethered ants with views of their natural foraging environment. The ants walk on an air-cushioned trackball, their movements are registered and can be fed back to the visual panorama. Views are generated in a 3D model of the ants’ environment so that they experience the changing visual world in the same way as they do when foraging naturally. The Antarium is a biscribed pentakis dodecahedron with 55 facets of identical isosceles triangles. The length of the base of the triangles is 368 mm resulting in a device that is roughly 1 m in diameter. Each triangle contains 361 blue/green LEDs and nine UV LEDs. The 55 triangles of the Antarium have 19,855 Green and Blue pixels and 495 UV pixels, covering 360° azimuth and elevation from −50° below the horizon to +90° above the horizon. The angular resolution is 1.5° for Green and Blue LEDs and 6.7° for UV LEDs, offering 65,536 intensity levels at a flicker frequency of more than 9,000 Hz and a framerate of 190 fps. Also, the direction and degree of polarisation of the UV LEDs can be adjusted through polarisers mounted on the axles of rotary actuators. We build 3D models of the natural foraging environment of ants using purely camera-based methods. We reconstruct panoramic scenes at any point within these models, by projecting panoramic images onto six virtual cameras which capture a cube-map of images to be projected by the LEDs of the Antarium. The Antarium is a unique instrument to investigate visual navigation in ants. In an open loop, it allows us to provide ants with familiar and unfamiliar views, with completely featureless visual scenes, or with scenes that are altered in spatial or spectral composition. In closed-loop, we can study the behavior of ants that are virtually displaced within their natural foraging environment. In the future, the Antarium can also be used to investigate the dynamics of navigational guidance and the neurophysiological basis of ant navigation in natural visual environments.

## Introduction

Ample experimental evidence now makes us confident that central-place foraging insects, such as ants, bees, and wasps navigate predominantly visually, relying on both scene memories and celestial compass information (e.g., Reid et al., 2011; Zeil, 2012; Collett et al., 2013; Wystrach et al., 2014; Graham and Philippides, 2017; Wehner, 2020). Visual navigation is supported by path integration (Heinze et al., 2018) which runs in the background, providing a failsafe, and in some cases and situations, also by olfactory, tactile and magnetic cues (Buehlmann et al., 2012, 2015; Knaden and Graham, 2016; Fleischmann et al., 2018). Evidence from behavioral studies and increasingly detailed knowledge of neural circuits relevant for navigation (e.g., Stone et al., 2017; Buehlmann et al., 2020; Kamhi et al., 2020; Steinbeck et al., 2020) are beginning to feed into neurally constrained and experimentally informed models of navigation (e.g., Baddeley et al., 2012; Ardin et al., 2016; Webb and Wystrach, 2016; Stone et al., 2017; Hoinville and Wehner, 2018; Gkanias et al., 2019; Schulte et al., 2019; Differt and Stürzl, 2020; Sun et al., 2020) and into robotic implementations (e.g., Lambrinos et al., 2000; Möller, 2000; Stone et al., 2016, 2017; Webb and Wystrach, 2016; Sabo et al., 2017; Dupeyroux et al., 2018).

The predictions of these models will likely become increasingly hard to test in behavioral experiments. The main reason being that controlled manipulations of complex visual cues, such as the full landmark panorama or conflict experiments between different compass systems are difficult to perform in natural navigation environments. Equally, investigations of the real-life computational properties of navigation-relevant neural circuits are currently hampered by limitations in the way visual information can be presented in electrophysiology rigs (see e.g., Table 1). There are currently no projection devices that can convey the full information content of the spatial, spectral, and polarization signal patterns that characterize natural navigation environments; and lastly the navigational competence of insects is based on active learning processes (e.g., Collett and Zeil, 2018; Jayatilaka et al., 2018; Zeil and Fleischmann, 2019) and relies on the active comparison between remembered and currently experienced input patterns (e.g., Zeil, 2012; Le Möel and Wystrach, 2020; Murray et al., 2020). It is thus likely that the neural machinery underlying navigation is heavily state-, context- and activity-dependent, requiring closed-loop control of the visual scene by the insect and control by the experimenter over the experience (What has been learned?), the motivation (What is the navigational goal?) and the state of the animal (Whether it holds information from path integration or not).

**Table 1:** Parametric comparison of existing insect research VR systems and the Antarium.

	Strauss et al. (1997)	Gray et al. (2002)	Lindemann et al. (2003)	Reiser and Dickinson (2008)	Takalo et al. (2012)	Paulk et al. (2014)	Koenig et al. (2016)	Kaushik et al. (2020)	Antarium
Colour	Green	RGB	Green	Green	White	Green	RGB	RGB	G, B, UV
Polarized	No	No	No	No	No	No	No	No	Yes (UV)
Technology	LED	Projector	LED	LED	Projector	LED	Projector + light guides	LCD	LED
Azimuth	±180°	±125°	±125°	*Depends*	±135°	±180°	±180°	±180°	±180°
Elevation	−0, +45°	±125°	−90°, +70°	*Depends*	−64°, +57°	−35°, +45°	−45°, +45°	−58°, +72°	−50°, +90°
Number of pixels	5,760	307,200	7,168	64 ·*N*	480,000	4,096	5,760	11 million	19,855 GB 495 UV
Intensity levels	2	256	8	8	256	2	256	256	65,536
Flicker (Hz)	1,000	60	00	372	360	>300	?	?	>9,000
Frame rate (Hz)	1,000	60	370	372	360	?	300	165	190
Angular resolution	20	<10	2.3°	*Depends*	a.so	3.5°	2–3°	0.14°	1.5° GB 6.7° UV
Light level	60 cd·m^−2^	14 lux	420 cd·m^−2^	*Depends*	4 W·m^−2^	168 lux	?	?	*N/A*
Closed loop	Yes	Yes	No	*Depends*	Yes	Yes	*Depends*	Yes	Yes

*A comparison of the properties of different comparable projection devices used in insect neuroethology work with those of the Antarium*.

With this in mind, we designed the Antarium, a panoramic projection device that would allow us to present ants walking on a trackball with views of their known foraging environment and to give the insects full control over the view transformations by feeding their intended movements back onto the panorama. Besides the engineering challenges of the device itself, there are two pre-conditions for this to work: a need to know the movements of the ants in their natural foraging environment and a way of reconstructing the views they will have encountered under natural conditions. To satisfy the first condition, we rely on several years of tracking ant movements with differential GPS, both during their normal foraging activity and after systematic displacement experiments (e.g., Narendra et al., 2013; Reid et al., 2013; Jayatilaka et al., 2014; Zeil et al., 2014). We second used LIDAR and camera-based methods to build 3D models of the ants’ foraging environment (e.g., Stürzl et al., 2015; Murray and Zeil, 2017), which we now can use to render panoramic views at any location within the foraging range of the ants and project them in the Antarium.

The Antarium is not the first “Virtual Reality” device in insect research but it is the first one that has been designed with the specific aim of enabling the presentation of natural, in contrast to synthetic, visual navigation environments (e.g., Van De Poll et al., 2015). We summarize the features of some devices described in the literature in Table 1 and briefly describe their properties below (see also Fry et al., 2004, 2008; Dombeck and Reiser, 2012; Schultheiss et al., 2017; Stowers et al., 2017).

Dickinson and Lighton (1995) built a cylindrical arena with green LEDs which was limited to display a dark vertical bar that could be rotated around the animal. The device could not display an arbitrary scene. Similarly, Strauss et al. (1997) designed a projector for walking *Drosophila* experiments. It is a cylindrical device, with monochrome (green) LEDs. A full-color computer projector with a hemispheric back-projected screen was built by Gray et al. (2002) and combined with a wind tunnel for moth research. The FliMax device (Lindemann et al., 2003) is an LED projector designed for fly research. It delivers a monochromatic (green) image for the tethered insect in its frontal visual field and was used to present reconstructed, outdoor view-sequences in electrophysiological experiments (Boeddeker et al., 2005). Reiser and Dickinson (2008) designed a modular projection device consisting of small identical square panels of monochromatic (green) LEDs. These modules can be used to tile a surface that has curvature around at most one axis, for example a cylinder^1^. The projection system designed by Takalo et al. (2012) is based on a modified video projector with elaborate optics. Paulk et al. (2014) used four LED panels to build a square well around the animal on the trackball. The panels are approximately 20 cm squares, with a 32 by 32 matrix of RGB LEDs on each. Only the green channel was utilized and only vertical bars were shown to the animal. Commercial projectors beamed onto a hemisphere were used by Peckmezian and Taylor (2015) who presented artificial 3D environments to trackball mounted jumping spiders. Koenig et al. (2016) projected simple shapes onto a rectangular array of light-guides, the other ends of which lined the walls of a cylindrical arena. More recently Kaushik et al. (2020) built an arena where the tethered insect is placed in the geometric center of a triangular prism formed by three high-speed commercial computer monitors turned on their side, delivering full-color video of a 3D modeled landscape.

The Antarium project aimed to design a projection system for experiments on ant navigation which must be capable of presenting panoramic views of the natural foraging habitat of ants in a way that addresses their spectral and polarization sensitivities while also allowing the ants to interact with the scene and the experimenter to modify it in arbitrary ways.

None of the existing projection systems could deliver on all these points. The following constraints were considered at the outset:

• Since ants have a panoramic vision (e.g., Zollikofer et al., 1995; Schwarz et al., 2011), the arena must cover 360° azimuth and the whole celestial hemisphere. Similarly, the arena must be able to project ground features down to −45° elevation.
• At the time the Antarium was designed, the spectral sensitivities of *Myrmecia* ants were not known, but scattered reports made it likely that ants, in general, possess UV, blue and green receptors (see references in Ogawa et al., 2015).
• The Antarium must be able to deliver light of sufficient intensities at these wavelengths. On a sunny day, the brightness in a natural scene can vary by 5 log units. The Antarium should be able to deliver a similar intensity range.
• Like most insects, ants possess a dorsal eye region with UV and polarization-sensitive receptors that feed into the skylight polarization compass system. The Antarium, therefore, would need to provide adjustable polarization covering the celestial hemisphere.
• We work with Australian bull ants. One of the largest bull ants (*Myrmecia pyriformis*) has around 3,500 ommatidia per eye (Narendra et al., 2011). Therefore, to avoid aliasing, the number of pixels must be at least 20 000.
• The critical flicker fusion frequency (CFFF) has been determined for two *Myrmecia* species, for the nocturnal *M. midas* at 84.6 ± 3.2 Hz and the diurnal-crepuscular *M. tarsata* at 154.0 ± 8.5 Hz (for review see Ogawa et al., 2019). For the Antarium, we opted for a minimum flicker rate of 300 Hz. The minimum frame rate for ants to observe continuous motion is not known, but it cannot be higher than the critical fusion frequency. Therefore, a frame rate close to 200 fps should be sufficient.
• We decided to use the trackball system designed by Dahmen et al. (2017) that records the rotations of a hollowed-out, air-supported Styrofoam sphere using optical mouse sensors. Besides a very high sampling rate, the advantages of this system are that it can be used in two ways: with the tethered animal free to rotate around the yaw axis and the trackball recording the animal’s translational movements only and with the tethered animal fixed, so that the trackball movements reflect both the yaw rotations and the translational movements of the animal.
• Finally, we had to operate within tight budgetary constraints.

The Antarium offers unique and crucial opportunities to investigate visual navigation in ants and to test models of visual navigation. It allows us to confront ants in both open and closed-loop with familiar and unfamiliar views of their natural environment, but also with completely featureless visual scenes, or with scenes in which dominant objects have been removed or displaced or that are altered in spatial or spectral composition. Most importantly, the Antarium can also be used in the future to investigate the neurophysiological basis of ant navigation in natural visual environments.

## The Antarium Design

### Geometry

Although an ideal projector would be spherical, several practical constraints make this untenable. For example, if LEDs were drilled and glued to the inside surface of a sphere, the optics would be ideal (see e.g., Koenig et al., 2016). However, hand-soldering thousands of LEDs to their driver is error-prone and extremely labor-intensive, and thus prohibitively expensive. A faster and cheaper alternative is to have machine printed circuit boards (PCB). PCBs can be any shape but must be flat, which constrains the projector to be a polyhedral approximation of a sphere. Since PCB manufacturing has a large NRE (non-recurrent engineering) cost, it is significantly cheaper if the polyhedron can be built from identical facets. Facet number is then a trade-off between optical properties and cost, with larger numbers leading to a better approximation of the sphere, but higher printing and labor costs. To guarantee that each facet has identical properties, i.e., that the LED arrangement can be identical on them, all of the polyhedron’s vertices should lie on a sphere.

**Figure 1:**
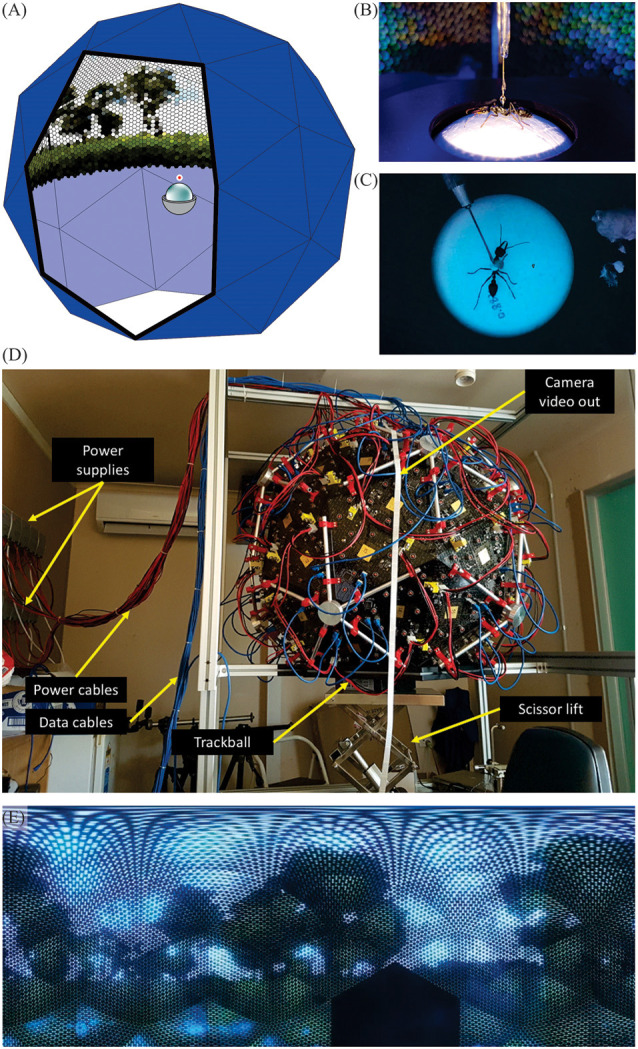
The Antarium. **(A)** Concept schematics of the biscribed pentakis dodecahedron with 55 facets of identical isosceles triangles carrying LEDs and control electronics and the trackball device. **(B)** Tethered ant on an air-cushioned trackball. The ants are free to rotate around the yaw axis, but its translational movements are registered by monitoring the rotations of the Styrofoam ball. **(C)** The tethered ant as seen by the Antarium camera. **(D)** The fully assembled Antarium. **(E)** The landscape panorama projected by the Antarium LEDs seen at 1.5° resolution, about twice the average resolution of ants.

We chose the biscribed pentakis dodecahedron (Figure 1A) as our spherical approximation for the Antarium. It has 60 facets of identical isosceles triangles. Five triangles form a pentagonal pyramid and 12 of such pyramids comprise the solid. For the Antarium one such pyramid is removed at the bottom, providing an opening where a trackball with the tethered animal can be inserted.

The physical size of the Antarium is constrained by electronic circuit board density, mechanical limitations, and the need for the opening at the bottom to be sufficiently large for the insertion of the trackball apparatus. With all those factors considered, the length of the base of the triangle was chosen to be 368 mm. All other dimensions are determined by the geometry of the pentakis dodecahedron, resulting in a roughly 1 m diameter device (Figure 1D).

### Pixel Arrangement

Ideally, the LEDs should be as evenly distributed on the surface of the polyhedron as possible, which is challenging, because the pattern continuity between adjacent panels needs to be addressed. A pattern was found where the LEDs are on the vertex points of a hexagonal lattice. A computer program was written that calculated the pixel positions and minimized the inter-pixel angle variation while taking the technological constraints of manufacturing into account.

Two such hexagonal grids were calculated, one for the GB (green/blue) pixels and another for the UV pixels. The angular acceptance functions are much wider and the spacing of ommatidia in the dorsal rim area is much higher than in the rest of the eye. It was decided that the UV LED pattern therefore should be made significantly sparser than the BG pattern, especially because of the high cost of UV LEDs and the need for their adjustable polarization.

**Figure 2:**
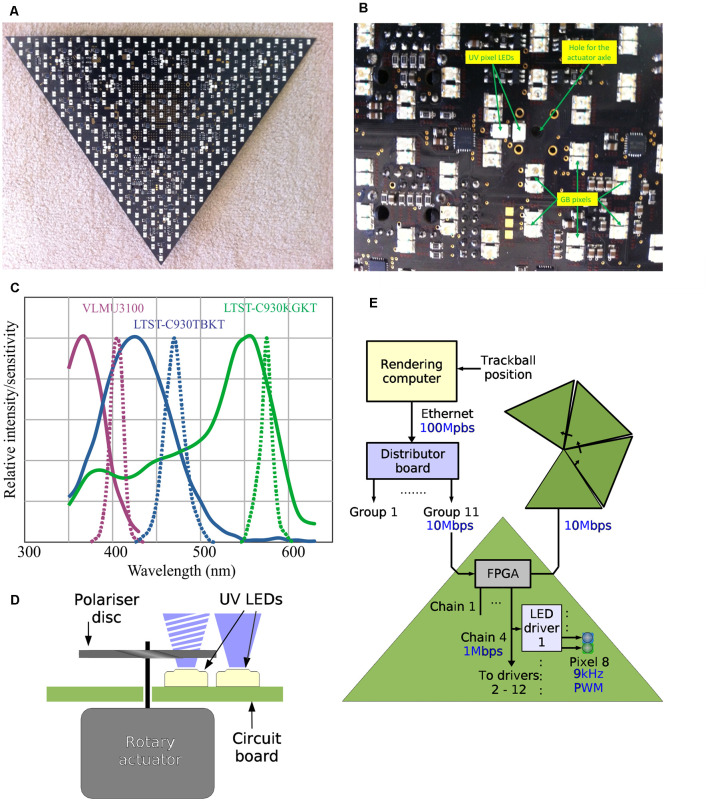
The design of individual Antarium panels. **(A)** Photograph of one of the panels with LEDs seen as white rectangles. **(B)** Detail of the panel LED locations on the printed circuit board and the actuator axle location for polarizer disks. **(C)** Spectral sensitivities of *Myrmecia* ants compared with current LED emission spectra. Continuous lines: normalized spectral sensitivities of the nocturnal *Myrmecia vindex* recorded intracellularly (redrawn from Ogawa et al., 2015). Dotted lines: emission spectra of the LEDs used in the current version of the Antarium as per manufacturer specifications. **(D)** Schematic of how light polarization is achieved. **(E)** The data path of the Antarium.

Each triangle contains 361 blue/green pixels and nine UV pixels (Figures 2A,B). Therefore, the 55 triangles that form the Antarium all together have 19,855 GB pixels and 495 UV pixels. Because no spectral sensitivity information was available at the time, the LEDs were chosen based on their price, availability, physical size, brightness, and beam angle. The selected LEDs were LTST-C930KGKT (Lite-On, Inc), LTST-C930TBKT (Lite-On Inc.,), and VLMU3100 (Vishay) for the green, blue, and UV, respectively. As can be seen in Figure 2C, the current LEDs’ spectral emissions are ill-matched to the photoreceptor spectral sensitivities that have since been determined in *Myrmecia* ants (Ogawa et al., 2015). This problem will be fixed in Antarium Mark II, which is currently under construction (see “Outlook” section below).

Preliminary experiments revealed substantial internal reflections within the Antarium, which were subsequently minimized by fitting a low reflection black cardboard cover to its internal surface. We measured the reflectance of the black cardboard with a USB-4000 Ocean Optics spectrometer against a certified reflectance standard reference from LabSphere illuminated by natural light. For all wavelength points, the cardboard intensity was divided by the reflectance standard’s intensity. Between 400 and 700 nm, the cardboard reflects between 5 and 7% of the light, without dips and peaks.

### Polarization

The adjustable polarization of the UV LEDs is based on each UV pixel being composed of two UV LEDs (Figure 2D). One of them is not polarized at all. The other one is placed behind a linear polarizer. The polarizer is a small disc mounted on an axle of a rotary actuator. The actuator can rotate the disc and therefore its plane of polarization can be at any angle. By varying the relative intensities of the polarized and unpolarized LEDs, the polarization depth can also be controlled.

The actuator needs to be fast as it must to be able to follow scene changes. Stepper motors and servos are too slow. The chosen actuator is an aircore, comprising of a small permanent magnet rotor and a stator with two coils arranged orthogonally. The combined magnetic fields of the two coils can have constant strength but set in any direction by driving one coil with a current that is proportional to the sine of the desired angular position while the other with its cosine. The permanent magnet rotor will always align with the magnetic field direction. Because the rotor is a low mass, an aircore can be driven into a new position quite fast. It has a tendency of oscillations while it settles, but manufacturers also offer devices with a small droplet of silicone oil in the rotor bearing. The oil acts as a damper and the time constant of the damping depends on the viscosity of the oil used. With the correct viscosity, the settling can approach the theoretical optimum. The chosen aircore, MicroAirCore 2022-715 from Simco, Limited was tested in the laboratory and it was fast settling, with very little oscillation. A 180° rotation can be achieved in less than 200 ms.

### LED Driving

To guarantee constant brightness the LEDs must be driven by a constant current source. The brightness of an LED is a function of the current flowing over it. LEDs are semiconductor diodes with nonlinear I–V characteristics. Also, as with all semiconductor devices, the characteristics are dependent on the temperature of the chip. Although a laboratory is usually an air-conditioned room, LEDs generate waste heat which warms them up. An LED that was bright for a while will be significantly warmer than one that ran at low intensity.

To mimic natural conditions, the intensity range of the arena should span close to 5 log units. A 16-bit linearly spaced intensity regime (65,536 levels) corresponds to 4.8 log units. We used a commercially available LED driver chip, the MBI5040 from Macroblock which satisfies all these criteria. It can drive 16 LEDs with a constant current. It uses a 16-bit pulse-width modulation (PWM) scheme to set the intensity of each LED individually. It can also apply a correction scheme to compensate for LED brightness variation. The correction scheme can vary the drive current from 0 to the nominal maximum in 1% steps for each LED separately. Also, it can detect and report short circuit and open circuit LED failures. Furthermore, the chip can operate with only a 0.5 V drop across its driving circuitry, an important feature from a power consumption point of view. The maximum drive current is 30 mA per LED; the LEDs used in the Antarium use only 20 mA drive current, far below the chip’s limits.

There are 361 BG and 9 UV pixels on a triangle and the MBI5040 can drive 16 LEDs (i.e., eight pixels), therefore each panel contains 47 chips.

### Flicker Considerations

Using PWM to set the LED brightness introduces flicker. PWM works by turning the LED full brightness for a short time then completely dark for some other time; the average intensity is the ratio of the ON time and the PWM period (the sum of the ON and OFF times). Thus, the LED flickers with the PWM period. Using discrete-time increments, the number of levels that can be displayed is the number of increments per PWM period. To ensure ants do not see the flicker, the Antarium needs a flicker frequency of 300 Hz or more. Thus, the PWM period needs to be no more than 3.33 ms which with 65,536 levels gives an elementary time increment of 50.86 ns, and a clock frequency of 19.7 MHz. We chose to run the PWM on a 20 MHz clock, even though the MBI5040 chip could run on up to 30 MHz.

However, another method allows us to reach a much higher flicker frequency far beyond what would be detectable by any biological system. The MBI5040 implements what is called scrambled PWM, a scheme designed to increase the flicker frequency above the PWM period. Instead of turning the LED on for the ON time then extinguishing it for the OFF time, the scheme spreads those times around within the PWM period. For example, if the period is 10-time units and the LED has a brightness of 30%, a simple PWM will turn it on for 3 units then off for 7 units. However, a scrambled PWM system might turn the LED on for 1 unit, then off for 2 units, on for 1, off for 2, on for 1, off for 3. Since the LED was on for 3 units and off for 7 the average brightness is still 30%, but now the LED blinked three times during the period instead of once. There are various ways to perform spreading. The MBI5040’s method becomes active when the brightness level increases above 32 units out of the 65,536. The Antarium uses a 20 MHz clock, thus if the LED brightness is higher than 0.05% of full scale, the flicker frequency will be more than 9 kHz, while below this threshold, for very dark LEDs, the flicker will be 305 Hz. Photodiode tests using an oscilloscope confirmed flicker at 9 kHz.

### Video Delivery and Frame Rate

Since the Antarium’s LED array is simply a display device, the method of data delivery from the rendering computer must be defined to understand all of the Antarium’s LED information. All together the Antarium has 20,350 pixels, each of which needs 2 × 16 bits of data to set the brightness, giving a total of 651,200 bits per video frame. The most common communication links on a computer are USB and Ethernet. When the Antarium was designed, the fastest USB was 450 Mbps (USB-2.0 full speed), the next step down was 12 Mbps (USB-2.0 high speed). The most common Ethernet interface was the so-called 100BASE-TX, delivering 100 Mbps over the ubiquitous "blue cable" (officially named Category-5 twisted pair cable). Full-speed USB interface chips were not readily available at the time and the high-speed USB was simply not fast enough. We, therefore, chose the 100 Mbps Ethernet link as the delivery medium for the video stream.

If a full-frame is 0.6512 Mbits, then the 100 Mbps link has a theoretical limit of 153 frames per second. In reality, it is less, as there are protocol overheads. That does not meet our goal of 200 fps and so we needed to find ways to compress the video stream.

The compression scheme must be relatively simple so that the panels of the Antarium can decode it and so that any computer can encode it without special hardware. The solution we chose is to subsample the color information. Instead of delivering 16-bit resolution green and blue values for a pixel independently, a 16-bit luminance value and an 8-bit chromaticity value can be delivered. That saves 25% of the video bandwidth (24 bits per pixel instead of 32). It does not compromise the 4.8 log unit brightness range, however, it does limit each pixel to 256 available hues.

The simplest way of sending data from a computer over an Ethernet link is by using a standard protocol that is supported by any operating system. One of those is UDP (user datagram protocol), where blocks of data (packets) are sent from one machine to another. UDP is advantageous in that it has a smaller overhead than other protocols. On the other hand, it does not guarantee delivery and gives no feedback on whether the packet ever arrived. UDP is often used in situations where the occasional loss of a packet is acceptable, but the unpredictable delays arising from confirming the reception of every packet and re-sending lost ones are not. These strengths and limitations are well suited for video streaming since if a single video frame gets lost, most of the time the observer will not even notice. Whereas if the streaming stopped while the sender and receiver negotiate the retransmission of a single packet, the video quickly becomes unwatchable. The Antarium, therefore, uses UDP for video delivery, with a dedicated Ethernet link to ensure that packet loss is rare.

An Ethernet frame contains up to 1,500 bytes of actual data (usually called the payload) and a further 38 bytes of addressing synchronization, and other ancillary information. Furthermore, UDP adds 24 bytes of protocol information to the data portion of the packet. The protocol overhead is thus 62 bytes for each Ethernet frame with a UDP packet in it. In a full video frame, a single Antarium triangle is represented by 1,110 bytes. Two extra bytes are added to the raw data, for reasons explained later. Therefore, the payload is 1,112 bytes. If each packet contains one triangle’s worth of video information, then 1,174 bytes need to be transferred per triangle. A video frame contains 55 such Ethernet frames, resulting in a maximum theoretical video rate of 194 fps over a dedicated Ethernet link. Indeed, in practice, the Antarium sustains around 190 frames per second.

### Architecture

Driving the nine polarisation actuators exceeds the capacity of available microcontrollers, so the Antarium’s panels are equipped with a field-programmable gate array (FPGA) instead. The processing unit of each triangle must receive video frames and send the brightness data to the 47 LED driver chips. Also, it must control the drive current of the nine actuators for the polarisers which each have two coils (18 total drive lines). Using pulse-width modulation (PWM) to set the current necessitates a device with 18 PWM units which no commercially available microcontroller can support. Instead, we chose to use an FPGA. An FPGA is just a large collection of simple digital logic building blocks, which then can be connected inside the chip to form a digital circuit that performs a specific function. Microcontrollers are well suited for tasks that work on fewer hardware signals at a time and where the decision making logic or calculations are complex. For tasks where there are many hardware signals and the calculations and decision making are relatively simple but must be performed at high speed and with precise timing, FPGAs are often a better choice. A large number of PWM signals make the FPGA a better solution for the Antarium. As such, each triangle panel contains an XC3S50AN chip from Xilinx, Incorporation. The chip has 50,000 logic gate’s worth of resources and can handle more than 80 input/output digital signals at high speed.

For our triangular panels, the FPGA needs to buffer a video frame, decode the compressed chromaticity, send the decoded data to the LED driver chips, and run 18 PWM controllers for the actuators, which consumes about 60% of its gates. The remaining 40% is not sufficient to also run Ethernet and UDP protocols as a logic circuit. While we could have used a more powerful chip, the added cost for every 55 panels would have been a significant expense. We instead chose to design a single interface board, with an associated one-off cost, that receives the video feed from the computer and distributes it to the triangles in a simpler way.

When the FPGA on each triangle panel receives a frame, it decodes the chromaticity encoding and collects the 16-bit intensity values for each LED in a buffer. At the end of the video frame, the buffer is sent to the LED driver chips. The drivers have an SPI (serial peripheral interconnect) interface, a standardized serial bus. The LED driver chips are designed to be daisy-chained. Since very long SPI chains are technically problematic, we divided the LED drivers into four chains. The FPGA delivers the video data to the chips on the four SPI chains simultaneously, which allows us to use a lower speed on the buses.

We use an H-bridge design for the PWM controller of the polariser’s actuators, which provides a large reduction in energy usage when the actuators are idle. To drive a single H-bridge the FPGA needs to produce two signals, so for the two coils of nine actuators each, 36 output signals are generated. This design allows energy to be saved since the FPGA reduces the current on both coils by the same factor (thus keeping their ratio, and therefore the angle of the actuator intact) when the actuator is stationary. This holding current is one-quarter of the current used for moving the actuator. If the actuator needs to be re-positioned, the FPGA switches the drive current back to nominal and when the position has not changed for a while, it slowly reduces the current to the one quarter holding value.

Finally, we placed thermal sensors on each triangular panel which are also controlled by the FPGA. The data from these sensors can be sent back across the network, which is important given the large amount of heat that can be produced when the full device is running at maximum brightness.

### Power Distribution

Since the Antarium consumes a significant amount of power, ensuring adequate power supply was integral. Each LED needs 20 mA for full brightness. A typical blue or UV LED has a voltage drop of around 3.4 V. The driver chip needs an extra 0.5 V, resulting in a minimum power supply voltage of 3.9 V. To cater for variations and to provide a safety margin, the LED driver circuitry operates from a 4.2 V supply. Due to the use of the intensity/chromaticity encoding, a pixel never needs more than 20 mA. Therefore, a triangle panel’s 370 pixels draw 7.4 A. Besides, the driver chips themselves also consume approximately 30 mA from the same supply. With 47 driver chips per panel that add 1.4 A to the load. The FPGA and its support circuitry need to be supplied as well, although that supply current is negligible compared to that of the LEDs and the drivers. The actuators run from 12 V and the nominal coil current is 54 mA. Due to the sin/cos driving scheme, however, the two coils of an actuator together have a maximum current consumption of 77 mA. The maximum current therefore is 0.7 A.

All together the board needs about 9 A from 4.2 V and 0.7 A from 12 V. The boards have two high-efficiency switch-mode power supplies that generate the 12 V and 4.2 V from a 24 V supply. The efficiency of these supplies is close to 90%, thus the board draws a maximum of 2.13 A from 24 V. Since under no circumstances will all LEDs of all triangles be on full power while all actuators being also set to their most power-hungry position, it was decided that a commercially available 24 V, 10 A power supply unit from MeanWell can safely power five triangles forming a pentagon. Eleven such units power the Antarium. Power losses on the cabling are minimized by using sufficiently thick wires.

### Thermal Considerations

The Antarium’s maximum power consumption is 2.5 kW, making its heat generation roughly equivalent to a portable oil radiator, enough to warm a small room with a volume of 16 m^3^. If that thermal energy were concentrated inside the Antarium’s less than 1 m^3^ volume, the temperature would rise to uncomfortably high levels for any subject very quickly. There are three ways to mitigate that risk: reducing the dissipated power, ensuring heat radiates outwards, rather than inwards, and ensuring convection between interior and exterior spaces.

Consumption is minimized due to our use of natural scenes, which are highly varied and contain many dark objects, such as trees trunks, buildings, and shadows on the ground (see Figure 1E). Furthermore, to compensate for the intensity variation due to parallax arising out of the Antarium’s geometry, the central area LEDs of each panel are artificially darkened. Together these two factors more than halve the overall power consumption.

Unfortunately, most of the heat is generated by the LEDs, which are on the inside of each panel. To minimize the amount of heat inside the Antarium we made use of the fact that each LED is connected to a solid copper plane near the outer surface of the PCB. While normally the thickness of copper in PCBs is 35 μm we used 70 μm copper for the Antarium to improve heat conductance. To further augment each panel’s heat conduction, we added a large exposed copper square to the exterior of each panel, which is thermally connected to the inner plane. This allows us to attach a Peltier cooling element with a heatsink and a fan, which can even more effectively suck the heat out and dissipate it. However, after testing the Antarium in its final form it turned out that there was no need for such additional cooling of the panels.

The lack of the need for a cooling element was perhaps facilitated by ensuring good airflow between the interior and exterior of the Antarium. This convection is assisted by a small table fan placed under the Antarium when it is operational, which supplies fresh air into the internal volume and forces the warm air out. Besides, an air-conditioned room helps to keep the internal temperatures at comfortable levels, and also ensures comfortable working temperatures for operators when set to 19°C.

We measured the temperature inside the Antarium at the position where the ant would be on the trackball using a Kestrel 5500 Weather Meter (Kestrel Australia, East Melbourne, VIC, Australia), the room air conditioning set at 19°C and after allowing temperatures to stabilize for 1 h. The temperature was recorded when it stopped changing over a 3 min period. We measured: Ambient room temperature: 20.5°C on a 26°C day; all LEDs on maximum output, no fan: 61.3°C; natural image, no fan: 28.3°C; natural image, with a fan: 25.1°C; ambient room temperature re-tested after the Antarium measurements: 20.5°C. This is well within natural foraging temperatures for both day- and night-active *Myrmecia* ants (Jayatilaka et al., 2011).

Figure 1D shows the fully assembled Antarium.

### Distributor Board

**Figure 3:**
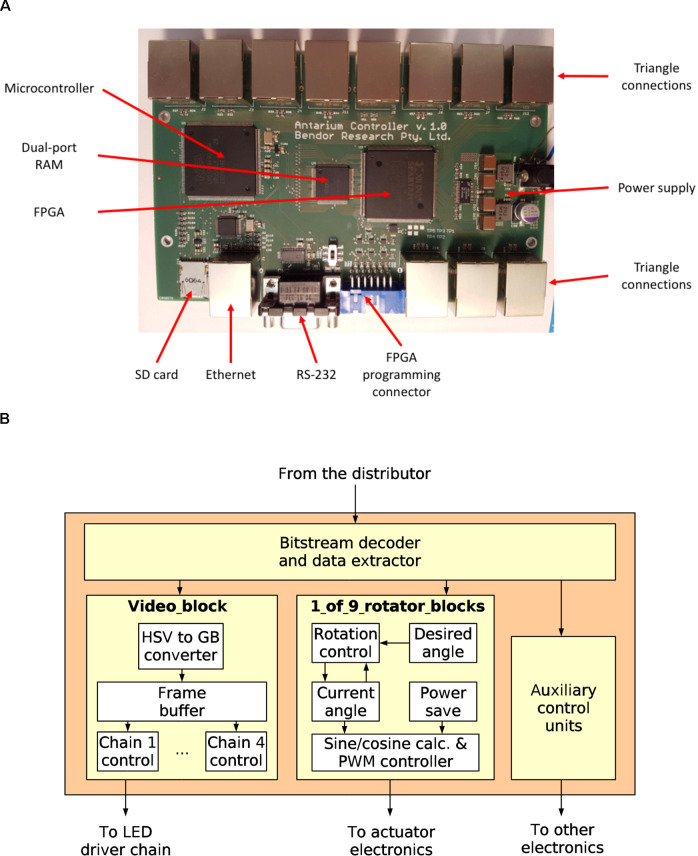
The Antarium control electronics. **(A)** The distributor board and its major electronics. **(B)** The block diagram of the LED panel Field-Programmable Gate Array.

The distributor board, as its name implies, distributes the video signal to the triangles (Figures 2E, 3). It contains an LPC1788 microcontroller from NXP, Inc. The microcontroller has an ARM Cortex-M3 core running at 120 MHz, 512 KB internal FLASH, and 96 KB internal RAM. It also has built-in peripherals, including an Ethernet protocol engine, an SD card protocol engine, several other serial communication blocks, timers, and user-programmable digital I/O ports. Its Ethernet engine, augmented with an external media access controller (TLK110, Texas Instruments) provides the 100 Mbps Ethernet interface.

The microcontroller shares its work with an XC3S500E (Xilinx, Inc.) FPGA containing half a million gates worth of logic. Between the microcontroller and the FPGA, there is a 128 KB dual-port static RAM chip (IDT70V28L, Integrated Device Technology). All received Ethernet frames are written into the dual-port RAM. Then the microcontroller decodes the protocol and analyses the packets. Packets related to connection maintenance are processed and responded to by the microcontroller. If the packet contains video data, then the microcontroller sends a message to the FPGA that the data should be delivered to a triangle. The FPGA examines the packet data, decides which pentagon it belongs to, and queues it for transmission on one of its 11 output links to the pentagons. After delivering the packet to the triangle the FPGA sends a message to the microcontroller informing it that the data are out and the given dual-port RAM region can be released.

If a triangle sends some data, then the FPGA holds the message in temporary internal storage, and after signaling the microcontroller that a message is available. When the microcontroller indicates that it is ready, the message is passed to it through the dual-port RAM.

The communication between the FPGA on the distributor board and the FPGAs on the triangles uses differential signaling. The data rate is 10 Mbps and the signal is subjected to the so-called Manchester encoding. That data speed and encoding are used by the 10BASE-T Ethernet standard, which facilitates the use of low-cost Ethernet connectors, magnetics, and cables. While the data speed and encoding method are the same, the protocol which the Antarium uses is much simpler than Ethernet. Each data frame starts with a preamble, followed by a synchronization byte, followed by a byte that indicates the type of the packet and its destination (or source) triangle within the pentagon. The next byte contains additional information about the packet content. The data follow and the packet is finished with a two-byte long data integrity check. That protocol is simple enough so that even the resource-limited FPGAs on the panels can handle it.

The configuration bitstream of the distributor board’s FPGA is stored on a micro-SD card. The board has an SD card socket and the microcontroller drives it. The controller implements the SD card protocol as well as the Microsoft FAT file-system, thus the FPGA bitstream can be written to the card using any computer. When the board is powered up, it first reads the SD card and loads the bitstream into the FPGA.

From the TCP/IP network stack, the firmware of the microcontroller also implements the UDP (user datagram protocol), IP (internet protocol), and ARP (address resolution protocol). Those are the necessary and sufficient components to be able to communicate with a machine with a standard network stack, regardless of the operating system it runs.

The distributor board also has a secondary function: to program the FPGAs on the triangles. The FPGA on the distributor board forgets its configuration when it is powered down. When the board is turned on, the microcontroller needs to load the configuration from the SD card. The FPGA on the triangle has built-in non-volatile storage to hold its configuration, thus it wakes up fully configured. However, the configuration first needs to be programmed into the non-volatile storage. Xilinx offers a free tool to do that, but the tool was slow and unreliable. Fortunately, the programming algorithm could be reconstructed from various application notes (engineering advisory articles). We then created our implementation of the algorithm on the distributor board and it can program the triangle’s FPGAs in a few seconds, with 100% reliability.

The distributor board is powered from a commercially available 12 V power module (plug-pack). The actual supply voltages for the electronics are generated from that 12 V using an LT3824 (Linear Technology) dual switch-mode regulator. To aid software development and the initial programming of the board also contains an RS-232 serial port.

### Design Tools

All design work was performed on a computer running the open-source GNU/Linux operating system. To aid engineering, several programs were written in-house to calculate or optimize certain parameters, to assist debugging, or to automate tasks. These programs were all written either in the C or in the Tcl language. Tcl/Tk is an open-source, interpreted scripting language with graphical capabilities. C programs were compiled using the open-source gcc toolchain. Building the final binary image or bitstream was controlled by the open-source gmake tool. The open-source Fossil distributed version control system was used to keep track of changes during development.

The schematic entry and the PCB design for the triangles and the distributor board were done using the commercial Eagle EDA package from CadSoft GmbH (recently taken over by Autodesk), version 6.4, professional edition, for Linux. The PCB manufacturing files were visually checked using the gebv open-source Gerber viewer tool.

The code for the FPGAs was written in the Verilog hardware description language. The logic simulations utilized the Icarus Verilog open-source simulator and the GtkWave open-source waveform viewer programs. Logic synthesis, technology mapping, place-and-route, and bitstream generation were performed by the ISE 14.7 toolchain from Xilinx, Inc. The tool is closed source but Xilinx provides it free of charge.

The firmware for the microcontroller on the distributor board was written in the C language. The code was compiled using gcc in a cross-compiler configuration. The open-source Armlib library from Bendor Research Pty. Limited was used for most low-level functions and the task scheduler. The Ethernet driver, SD card driver, and the FAT filesystem utilized routines donated by Arthur Digital Solutions Kft (Hungary).

The component sourcing, purchasing, PCB manufacturing, and assembly were ordered from Albacom Kft (Hungary). Quality control and thorough testing of the boards before shipment to Australia was performed, gratis, by Arthur Digital Solutions.

The mechanical design and the manufacturing of the scaffolding were done by the ANU workshop. The power cables were manufactured by hand; the Ethernet cables, wires, and sundry electronics items were purchased from Jaycar, a local electronics store.

### 3D Rendering and Driver Software

The software that generates the video stream for the projector makes use of the commercially available three-dimensional (3D) rendering engine Unity (Unity Technologies) running in Microsoft Windows^@^. The primary market for the engine is computer games and as such it is best suited for planar projections. The Antarium has a low pixel count compared to most commercial video games and it is, therefore, possible to render six or more game views simultaneously at a high frame rate, on modern graphics cards. The six views have the same camera position in the 3D virtual world, but the cameras look in six orthogonal directions (up, down, left, right, front, and back), essentially creating a projection onto a cube. A custom shader uses a spherical transformation known as cube-mapping to map the pixels of our rendered cube onto any arbitrary 3D model. By applying this shader to a 3D model that represents each LED in the Antarium as an individual face, with the same azimuth and elevation as the LED’s real-world coordinates, we can render the scene as it would appear if projected onto the Antarium. We then use a compute shader to sample each face of our virtual Antarium using its normal as a lookup into the now spherical cubmap (using DirectX SampleLevel function). Finally, we encode and package these as pixel data to send over UDP to the distributor board.

The Antarium aims to display views of the natural habitat of the animals (Figure 1E). We, therefore, constructed a 3D model of that habitat using camera-based reconstruction methods (see Stürzl et al., 2015; Murray and Zeil, 2017). Thousands of photographs were taken with a Panasonic Lumix DMC-FZ200 camera at 4,000 × 3,000 pixel resolution while walking around in the area surrounding the nests of the experimental ants. Multiple voxel clouds were created from these photographs with the software Pix4D (Pix4D SA) and exported as 3D models before being combined into a single unified and aligned 3D reconstruction of the ants’ foraging environment. Since the very distant panorama does not have enough parallax to be processed by the 3D reconstruction software, we added the distant panorama later as a static background image at 1 km (approximately infinite) distance. We captured this panorama with a Ricoh Theta S panorama camera (Ricoh Company Limited, Tokyo, Japan).

This procedure allows us to capture views from within our 3D model, or from within projections of panoramic photographs, to edit the 3D model (using Blender) or photographs to fix errors (using Paint.net), and finally to generate experimental treatments (using Unity3D). For example, *Myrmecia* ants regularly visit trees for foraging (e.g. Narendra et al., 2013; Reid et al., 2013; Jayatilaka et al., 2014) and we are now able to extract such foraging trees from the photograph and the 3D model, allowing us to move the foraging tree to any arbitrary location or bearing in the model/photograph as an ant is viewing the scene inside the Antarium. We can then ask, whether the ants treat trees as individual landmark beacons, or get their bearing from the whole landmark panorama.

### The Trackball System

The ants are placed on an air-cushioned, light-weight, 10 cm diameter trackball (Figures 1B,C) on which they are free to rotate around the yaw axis but that allowed us to record their intended translational movements as described in detail by Dahmen et al. (2017; see also Murray et al., 2020). The trackball sends the position data to the rendering computer using USB. In a departure from the original, we now maintain and compile the trackball code using Microsoft Visual Studio in the C language (Microsoft Inc. 20XX). The USB connection relies on the open-source usblib library. The system response is linear up to speeds of 1.2 m/s (for detailed system properties see Dahmen et al., 2017).

Since the trackball is connected to the computer running the 3D engine, we can use the movement data it generates to update the position of our virtual cameras in the 3D world, thus providing our ant subjects with closed-loop control of the visual scene. When running in an open-loop, 3D scenes or panoramas can be presented either statically or in sequence. For closed-loop, we use Kernel32 to share a file in shared memory between the trackball program and the game engine. In this file, we write the current offset of the trackball from its starting location and accept commands to reset the starting location, such as when a new treatment begins. In both modes, the human operator, or their code, can arbitrarily change the ant’s virtual position and heading at any time. However, in the closed-loop mode, this trackball offset can be used to update the position of the six cameras inside the 3D model, thus updating the view that is presented to the ant subject, based on its movement on the trackball. It should be noted that due to the complexity of this setup significant care must be taken to ensure all real-world and virtual objects are rotationally aligned so that the visual consequences of the ant’s movements are accurately represented.

### Antarium Camera

To record in addition to the ants’ intended paths also the scanning movements of their head, we mounted a Raspberry-Pi V1 camera at the apex of the Antarium. The camera is connected to a Raspberry-Pi single-board computer (Raspberry Pi Foundation, UK). It records a 1,280 × 960 pixel video at 30 fps to an external USB disk (Figure 1C). The recording format cannot be played back with commercially available software on Windows, thus the recorded footage is transcoded to MP4 format using the open-source ffmpeg package on a Linux computer.

### Proof of Concept

**Figure 4:**
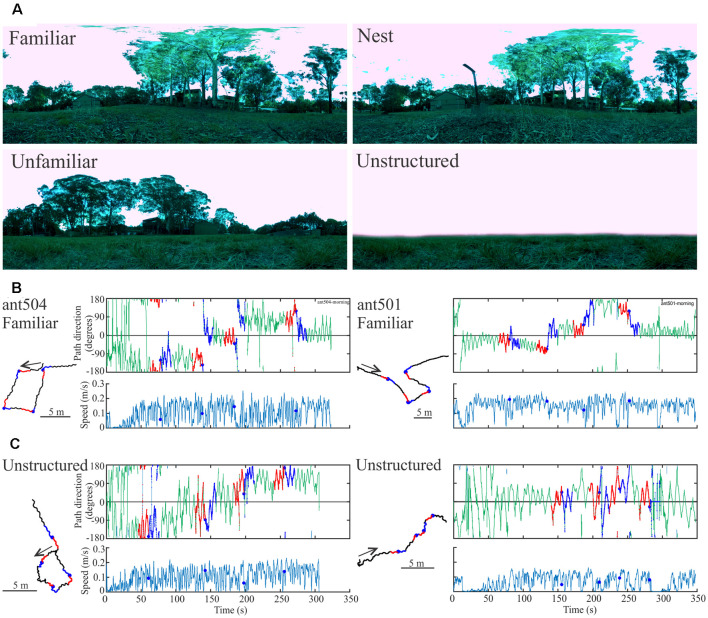
Proof of concept experiments. **(A)** Four panoramic views from the ants’ foraging habitat. *Familiar* is located on the ants’ foraging corridor, half-way toward their foraging tree; *Nest* is the view from the ants’ nest entrance; *Unfamiliar* if the view from a location about 5 m to the side of the foraging corridor and *Unstructured* is a synthetic view without landmark panorama. **(B)** Two examples (left and right) of ants responding to familiar scene rotations. Instances of rotations are marked by blue dots in the time course of path direction (top) panels and of speed (bottom panels). Fifteen seconds segments before (red) and after rotations (blue) are also marked on the intended paths of the ants (shown on the left) and on the time course of path direction (top panels). Paths are shown in the trackball coordinate system. **(C)** Same as **(B)**, but in the presence of the unstructured scene. Note the difference in path direction oscillations in **(B,C)**.

To date, we have conducted several experiments demonstrating that ants recognize familiar scenes in the Antarium and derive navigational instructions from them. We will present these behavioral results in a separate publication. In brief, we confronted ants tethered on the trackball with four different views (Figure 4A): a familiar view half-way toward a tree along their normal foraging corridor (*Familiar*), the view from the nest (*Nest*), an unfamiliar view from a location about 5 m offset from the foraging corridor (*Unfamiliar*) and a scene that consisted of a horizon line only (*Unstructured*). As the ants walked on the trackball in these four situations, we instantaneously rotated the scenes several times through 90 degrees randomly clock- or counter-clockwise to test whether the insects took note of panorama information. They indeed changed path direction in response to such rotations when confronted with any of the structured, but not the unstructured scenes as shown for two examples of the Familiar scene in Figure 4B (*Familiar*) and Figures 4C (*Unstructured*), with 15 s long segments before rotations labeled red and 15 s segments after rotations labeled blue. Instances of rotations are marked by a blue dot. Note that the ants’ speed is not constant, but indicates that the ants move in spurts (Figures 4B,C) and that their path direction oscillates with smaller amplitudes when confronted with a familiar scene and larger amplitudes when confronted with an unstructured scene.

**Figure 5:**
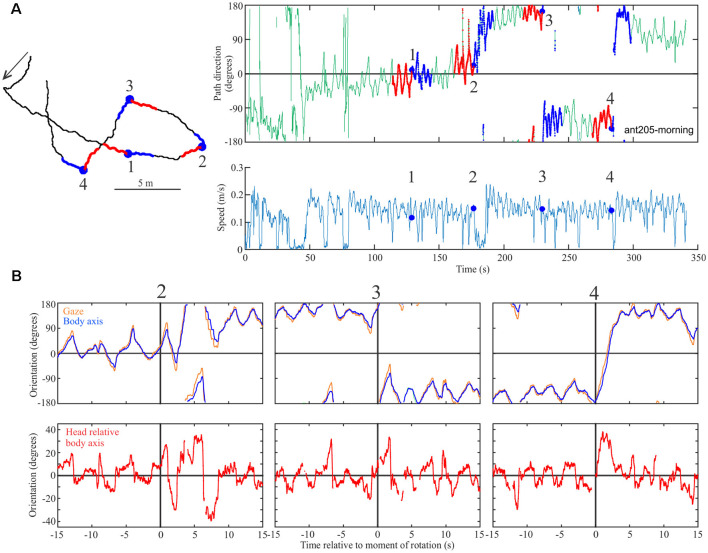
Proof of concept experiments. **(A)** The path (left), the time course of path direction (right-top), and time course of speed (right bottom) for an ant in the presence of the familiar view. Successive instances of scene rotation are marked by blue dots and numbered. Otherwise conventions as in Figure 4. **(B)** Top row: gaze (head, orange) and longitudinal body orientation (blue) over time from 15 s before and 15 s after rotation 2–4. Bottom row: head orientation relative to longitudinal body axis for the same segments. The vertical black line marks the moment of rotation.

For another example of responses to the familiar scene rotations (Figure 5), we extracted the head- and longitudinal body axis orientation of the ant from the Antarium camera footage 15 s before to 15 s after the rotation (Figure 5B). Following rotation, the ant’s head- and body scanning movements tend to increase (Figure 5B) as she changes her heading direction in the three instances in which she responded to the rotation.

## Outlook

The Antarium is a unique reconstructed visual reality arena for ants. No projection system before it has offered a completely panoramic projection tuned to an insect’s vision, including arbitrary polarization patterns. Furthermore, the Antarium can deliver accurate recreations of the visual reality of animals, by projecting imagery captured from their natural habitat rather than artificially generated scenes (e.g., Stowers et al., 2017; Kaushik et al., 2020). We see the ability to present natural views that are familiar to an insect as an important condition for answering many questions about the neural mechanisms underlying visual navigation.

The Antarium not only allows us to compare responses to familiar and unfamiliar natural scenes, but we can also add, remove or dislocate landmarks, set up conflicts between different visual information (i.e., celestial vs. terrestrial), and manipulate the intensity, the color, or the spatial frequency composition of scenes. In closed-loop, we can investigate the dynamics of visual navigation, such as the relationship between navigational decisions and scanning movements, or the frequency with which ants check and update their heading direction.

Since the initial conception of the Antarium, many advancements have been made, both in the development of LEDs and in our knowledge of the neural and visual systems of ants. These advancements combined with lessons from our experiments with the Antarium, have led us to design a second version, the Antarium Mark II to improve upon the original. For instance, we now know that the spectral sensitivities of *Myrmecia* photoreceptors in both day- and night-active species have peak sensitivities around 375, 430, and 550 nm (Figure 2C; Ogawa et al., 2015). As LEDs with expanded emission in the UV range have become available and have dramatically decreased in cost, we can now much more precisely match LEDs to ant spectral sensitivities and increase the density of UV LEDs. Antarium Mark II will thus provide much-improved UV contrast of the landmark panorama, which has been shown theoretically and in behavioral experiments to be important for providing information on heading direction (e.g., Möller, 2002; Kollmeier et al., 2007; Graham and Cheng, 2009; Stone et al., 2014, 2016; Differt and Möller, 2015; Schultheiss et al., 2016).

## Data Availability Statement

The raw data supporting the conclusions of this article will be made available by the authors, without undue reservation. For design share and construction options contact ZK (zoltan@bendor.com.au).

## Author Contributions

ZK: developed design, architecture, electronics and software; supervised manufacturing; conducted tests and behavioral experiments; and wrote the first draft of the manuscript. TM: conducted 3D modeling and developed rendering software and interface pipeline to the Antarium; conducted tests and behavioral experiments. HD: provided trackball system and training. AN: conducted initial experiments with a trackball system and provided funding. JZ: project supervision and provision of funding. All authors contributed to several revisions of the manuscript. All authors contributed to the article and approved the submitted version.

## Conflict of Interest

ZK is a director of Bendor Research Pty. Limited, an embedded systems consultancy company. To keep cost low, the company’s existing contacts were utilized in the component sourcing and manufacturing of the Antarium and its test equipment was used during development. Bendor Research has not charged any fees for its services and had no gain, financial or otherwise, from providing them. The remaining authors declare that the research was conducted in the absence of any commercial or financial relationships that could be construed as a potential conflict of interest.

## Footnotes

^1^A bi-colour (green and blue) version of that device is now commercially available and a trichromatic (green, blue, and UV) device is being designed (Michael B. Reiser, personal communication).

## Abbreviations

FPGA: field-programmable gate array
PWM: pulse-width modulation
PCB: printed circuit board
SPI: serial peripheral interconnect
UDP: user datagram protocol.
